# Regulation of T Cell Immunity in Atopic Dermatitis by Microbes: The Yin and Yang of Cutaneous Inflammation

**DOI:** 10.3389/fimmu.2015.00353

**Published:** 2015-07-13

**Authors:** Tilo Biedermann, Yuliya Skabytska, Susanne Kaesler, Thomas Volz

**Affiliations:** ^1^Department of Dermatology and Allergy, Technische Universität München, Munich, Germany; ^2^Department of Dermatology, University Hospital, Tübingen, Germany

**Keywords:** atopic dermatitis, bacteria, dendritic cells, microbiota, non-pathogenic, T cell, tolerance, skin

## Abstract

Atopic dermatitis (AD) is a chronic inflammatory skin disease predominantly mediated by T helper cells. While numerous adaptive immune mechanisms in AD pathophysiology have been elucidated in detail, deciphering the impact of innate immunity in AD pathogenesis has made substantial progress in recent years and is currently a fast evolving field. As innate and adaptive immunity are intimately linked, cross-talks between these two branches of the immune system are critically influencing the resulting immune response and disease. Innate immune recognition of the cutaneous microbiota was identified to substantially contribute to immune homeostasis and shaping of protective adaptive immunity in the absence of inflammation. Disturbances in the composition of the skin microbiome with reduced microbial diversity and overabundance of *Staphylococcus spp*. have been shown to be associated with AD inflammation. Distinct *Staphylococcus aureus* associated microbial associated molecular patterns (MAMPs) binding to TLR2 heterodimers could be identified to initiate long-lasting cutaneous inflammation driven by T helper cells and consecutively local immune suppression by induction of myeloid-derived suppressor cells further favoring secondary skin infections as often seen in AD patients. Moreover dissecting cellular and molecular mechanisms in cutaneous innate immune sensing in AD pathogenesis paved the way for exploiting regulatory and anti-inflammatory pathways to attenuate skin inflammation. Activation of the innate immune system by MAMPs of non-pathogenic bacteria on AD skin alleviated cutaneous inflammation. The induction of tolerogenic dendritic cells, interleukin-10 expression and regulatory Tr1 cells were shown to mediate this beneficial effect. Thus, activation of innate immunity by MAMPs of non-pathogenic bacteria for induction of regulatory T cell phenotypes seems to be a promising strategy for treatment of inflammatory skin disorders such as AD. These new findings demonstrate how detailed analyses identify partly opposing consequences of microbe sensing by the innate immune system and how these mechanisms translate into AD pathogenesis as well as new therapeutic strategies.

## Introduction

The skin is the body’s outer interface organ forming a multi-layered barrier between the environment and the individual. To maintain integrity of the host and protect from potentially detrimental influences by pathogens or toxic substances, effective defense mechanisms have evolved. In addition, the skin is colonized by a multitude of bacteria forming the cutaneous microbiome that provides essential functions for skin homeostasis. Thus ­anti-bacterial immune responses must be tightly controlled to allow pathogen defense but also to preserve the composition of the microbiota in the absence of inflammation. Innate and adaptive immunity interact and orchestrate different qualities of immune responses. A breakdown in the symbiotic relationship between the cutaneous microbiota and its host has been identified and is associated with skin inflammation in atopic dermatitis (AD) (1). Deciphering mechanisms of the innate immune system which promote skin inflammation in AD patients and identifying counter regulatory pathways that limit inflammation by shaping T helper cell responses have not only broadened our understanding of disease pathology but also opened up new therapeutic perspectives.

## Atopic Dermatitis Pathogenesis: In the Beginning, It is All About T Cells

Atopic dermatitis is a frequent inflammatory skin disease affecting up to 10–20% of the children and approximately 3% of adults in western countries (2). Innate (mast cells, eosinophils, basophils, different types of dendritic cells (DC), innate lymphocytes, and myeloid-derived suppressor cells) and adaptive immune cells (B cells and T cells) are believed to contribute to the complex immune network underlying cutaneous inflammation in AD. Few of the experimental setups and studies trying to disclose a role for these different cell types for AD allow defining one of these cell types as causal for cutaneous inflammation with the exception of CD4^+^ T helper cells which have been shown in several studies, clinical trials, and mechanistic analyses to drive AD (3–5). Consequently, a closer look at the state of the art in regard to T cells in AD is essential to create a modern concept of AD pathogenesis and to develop new therapeutic strategies.

**Figure 1:**
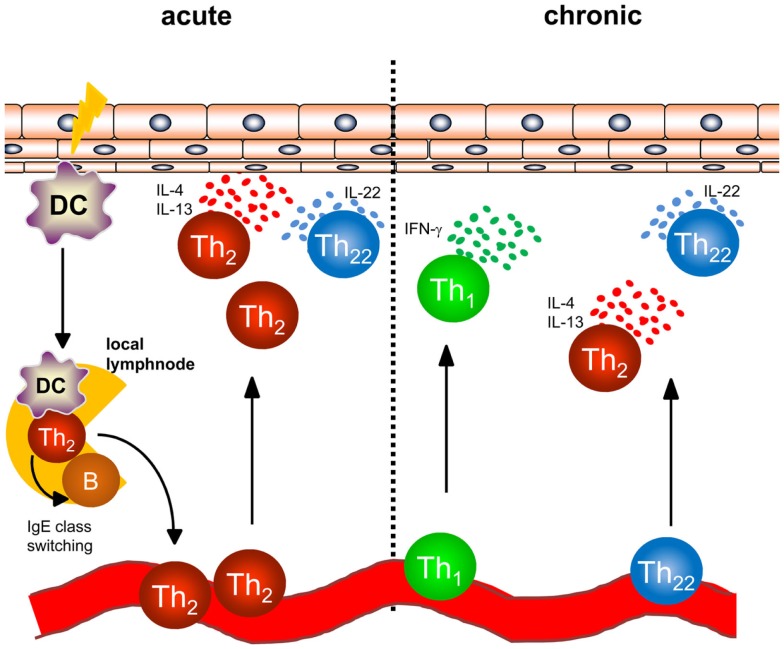
**Overview of T helper cell populations in acute and chronic dermatitis**. In acute atopic dermatitis activated skin-resident DC migrate to local lymphnodes to prime naïve T helper cells and polarize them into a Th2 phenotype. Th2 cells induce IgE class switching in B cells accounting for enhanced IgE levels regularly found in atopic dermatitis patients. Th2 cells are recruited back to the skin and induce cutaneous inflammation by effector cytokines accompanied by Th22 cells. In chronic atopic dermatitis, Th1 cells are increasingly part of the skin infiltrate consisting of Th1, Th2, and Th22 cells.

In AD, a dense infiltrate of activated CD4+ T cells can be detected in the dermis especially in acute lesions (Figure 1) (6). To better understand the initiation of AD, analyses of atopy patch test lesions has contributed substantially. Analyses of cytokine expression revealed that T helper cells of early lesions produce IL-4, IL-5, and IL-13, hallmark cytokines of Th2 cells (7). Thus, the concept that was developed within the last 20 years was based on the interpretation that Th2 cytokines in the skin promote cutaneous inflammation in AD. As examples of Th2-associated pathology, the Th2-induced isotype switch in B cells leading to the production of IgE is frequently cited and IL-5 promoting maturation and survival of eosinophils are highlighted to play a role in some types of AD and other atopic diseases (8, 9). Mechanistic analyses have helped to understand how these Th2 cells are recruited to the skin also disclosing possible targets of new therapeutic strategies: cutaneous leukocyte antigen (CLA) as adhesion molecule allowing Th2 cells to roll on the luminal side of the high endothelial venules proved to be a good marker for T cells prone to migrate to the skin (10, 11). CCR4 and CCR10 were shown to be prominent chemokine receptors allowing T cells to migrate through endothelia in the skin upon binding with the respective chemokines such as CCL17, CCL22, and CCL27 (11–13), and the chemokines binding CCR4 are among the best biomarkers for AD inflammation (14). Once Th2 cells are recruited to AD skin, Th2 cell activation leads to the accumulation of high levels of Th2 cytokines. Interestingly, IL-4 orchestrates monocytes and DC to produce high amounts of CCR4-binding chemokines further amplifying Th2 cell recruitment to the skin (15–17). Besides the effect on these immune cells, Th2 cytokines also fundamentally influence keratinocytes and how the epidermis responds to different stimuli. Th2 cytokines suppress the expression of terminal differentiation proteins of keratinocytes (filaggrin, loricrin and involucrin) therefore destabilizing cutaneous barrier function. In addition, Th2 cytokines have been shown to suppress the upregulation of antimicrobial peptides (AMP) in the skin upon stimulation such as beta defensin (HBD)-2, HBD-3, and LL-37 in keratinocytes. This failure to secrete AMPs to reach levels found in psoriasis has been argued to be the underlying mechanism of the susceptibility to bacterial and viral infections found in AD (18–20). Bacterial colonization on AD skin may be further supported by IL-4-mediated enhanced expression of fibronectin and fibrinogen acting as adhesion molecules for *Staphylococcus aureus* (21, 22). The Th1 cell and Th17 cell subsets are known for their potent anti-infectious properties controlling for intracellular and extracellular bacterial and fungal infections (23). Thus, demonstrating that IL-4 potently suppresses Th1 and Th17 cell immunity (24–28) further emphasized that AD skin is fundamentally more susceptible to cutaneous colonization and infection than normal or psoriasis skin. Most recent analyses even demonstrated that IL-4 reduces the Th17 inducing and maintaining cytokine IL-23 in antigen presenting cells both *in vitro* and *in vivo* in humans (28). These findings highlight that analyzing the recruitment, persistence, and function of different Th cell subtypes into AD skin is of pivotal importance for better understanding AD and for disclosing the impact of bacteria for AD inflammation, its prevention, and resolution.

Th17 cells were characterized by the production of IL-17 and IL-22 (29, 30). Following Th17 characterization, screening analyses were carried out for different diseases and tissues to better understand the Th17 cell function. Immunohistochemical studies revealed IL-17 production in acute AD lesions and confirmatory studies showed correlation of AD severity with the number of IL-17-producing T cells in peripheral blood and acute lesions (31, 32). Further characterization of IL-17-producing T cells in acute AD lesions revealed that IL-17 was produced by newly described subsets of Th2/IL-17^+^ and Th0/IL-17^+^ cells (33). Interestingly, IL-17 production by these subsets required stimulation by staphylococcal superantigens indicating interdependence of bacterial products and IL-17 in AD skin. It is still not understood why despite Th2 cytokines such as IL-4 suppressing IL-17 and IL-23, IL-17-producing cells are still detected in AD and whether IL-17 contributes to AD initiation or represents an epiphenomenon of cutaneous colonization and infection with bacteria in AD (27, 28, 33). Thus, the role of IL-17 in AD needs further clarification and new drugs being available targeting IL-17 and IL-17R for the treatment of psoriasis such as secukinumab will soon shed light into the hitherto unknown role of IL-17 for AD. As microbiota also induce or condition for IL-17 production, defining the role of IL-17 for skin homeostasis, defense, and inflammation requires functional analyses, disclosure of the cellular network, and spatiotemporal differentiation.

More recently, another unique subset of T helper cells enriched in inflamed human skin producing IL-22 in the absence of IL-17 was identified and characterized (34). These Th22 cells express the skin homing chemokine receptors CCR4 and CCR10 like Th2 cells and are distinct from Th17 cells as shown by transcriptome analyses (34, 35). Accumulation of Th22 cells was demonstrated in acute and chronic lesions of AD (36, 37) as were IL-22-producing CD8+ T cells (33, 36). IL-22 binds to a complex of IL-22R1 and IL-10R2 for induction of downstream signaling (38). IL-22R1 is not expressed on hematopoietic cells but rather can be detected on tissue-resident cells including keratinocytes (39). Functional consequences of IL-22 production are dependent on the target organ and the presence or absence of other cytokines, such as IL-17 or TNFα leading to either protective immune responses or inflammation (38, 40). IL-22 acting on keratinocytes has been reported to downregulate filaggrin expression and to affect expression of profilaggrin processing enzymes leading to further impairment of the epithelial barrier (41). Furthermore, IL-22 was reported to inhibit terminal differentiation of keratinocytes and to induce epidermal hyperplasia which is prominently seen in chronic AD (34). Thus IL-22-producing T cells may well play a crucial role in the pathogenesis of AD (42), however, the exact role cannot be defined based on the data available. Functions enhancing processes of both inflammation and regeneration describe Th22 cells as a Janus-like player for AD.

## Atopic Dermatitis Pathogenesis: Chronic and Self-Perpetuating Inflammation through Bacterial Exposure

While acute flares of AD are characterized by an infiltrate consisting of Th2 and Th22 cells, Th1 cells can be detected in chronic lesions of AD (Figure 1) in addition to Th2 and Th22 cells as early as 48 hours following elicitation of dermatitis (7, 37, 43). Th1 cells are characterized by the transcription factor T-bet and the secretion of the inflammatory cytokine IFN-γ (44). In regard to the pathogenesis of chronic AD, IFN-γ was postulated to contribute to skin hypertrophy in chronic AD (45). IFN-γ strongly induces IL-22R1 on keratinocytes allowing IFN-γ and IL-22 to act together for induction of epidermal hyperplasia (39).

**Figure 2:**
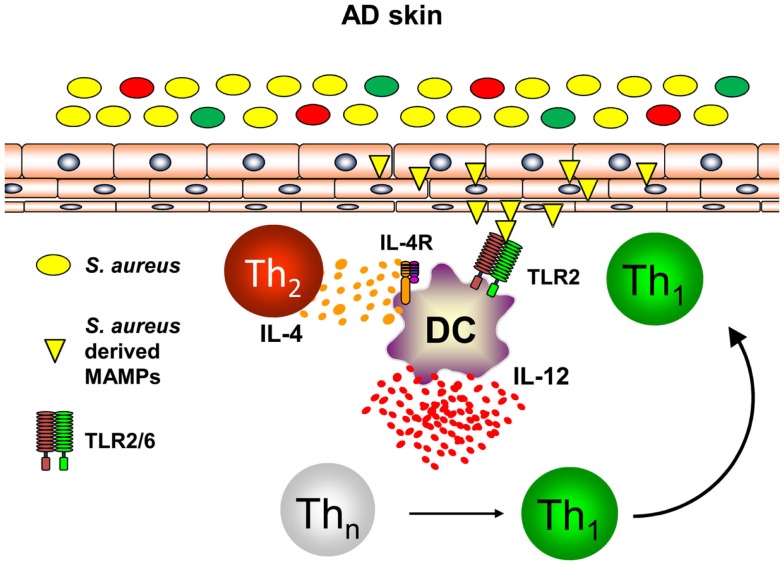
**Dual activation of skin-resident DC by IL-4 and TLR2 ligands promotes IL-12 expression and Th1 polarization**. In atopic dermatitis skin, Th2 cells secreting IL-4 are abundantly present. Skin-resident DC are activated by *S. aureus* derived TLR2 ligands (lipoproteins, lipoteichoic acid) in an IL-4 rich environment leading to DC maturation and enforced IL-12 secretion by combinatorial activation of TLR2- and IL-4R-signaling while IL-10 production is markedly attenuated. As a consequence in the local lymph nodes, naïve T helper cells are preferentially polarized into a Th1 phenotype promoting long-lasting cutaneous inflammation after homing to atopic dermatitis skin.

Th1 cells can be polarized from naïve T helper cells by DC secreting large amounts of IL-12p70 (46, 47). As DC are at the interface of innate and adaptive immunity and build a dense network of immune sentinels in the skin, innate immune signals activating skin-resident DC were postulated to contribute to enhanced priming of Th1 cells in AD leading to chronic skin inflammation (48, 49). *S. aureus* colonization found in the majority of AD skin lesions and very early during lesion development has been shown to contribute to the release of pharmacological relevant amounts of Toll-like receptor (TLR2) agonists such as lipoteichoic acid (50). Binding of *S. aureus-*derived lipoteichoic acid to TLR2 on DC *in vitro* leads to DC maturation and production of the pro-inflammatory cytokines IL-12p70 and IL-23 resulting in enhanced Th1 and Th17 priming (51). Amplification of Th1 polarization is further achieved by the presence of the Th2 cytokine IL-4 acting on DC resulting in enhanced IL-12p70 production during T cell priming (52–54). As IL-4 is abundantly present in the skin of acute flares of AD as is *S. aureus*, combinatorial activation of DC by IL-4 and TLR2-ligands is a constant feature in AD skin. Based on this combined stimulation of cutaneous DC, a profound shaping of consecutive immune response can be anticipated (55). Indeed, TLR2 activation together with IL-4R signaling transfers acute Th2-driven dermatitis (48 h before dermatitis resolution) into long-lasting skin inflammation (14 days) with enhanced expression of the Th1 cytokine IFN-γ (56). A key role could be ascribed to the Th2 cytokine IL-4 mediating suppression of the anti-inflammatory cytokine IL-10 while enhancing IL-12 (Figure 2). Administration of exogenous IL-10 reverted chronic skin inflammation induced by TLR2 ligands and IL-4 indicating a crucial role for sustained production of IL-10 in response to cutaneous exposure to microbes. In conclusion, innate immune signals derived from *S. aureus* colonization or infection play a pivotal role in the transition of acute dermatitis into chronic skin inflammation and disease exacerbation highlighting a crucial role for microbes in the pathogenesis of AD (56). Furthermore, these mechanistic studies in AD models also revealed that IL-4 is responsible for the transition of early self-limiting AD into chronic self-perpetuating AD as found in most of AD patients. These findings demonstrate that Th2 cytokines such as IL-4 and IL-13 are not only responsible for early AD lesions but also for the development and maintenance of chronic AD. This also explains why targeting AD by the recently described antibody dupilumab blocking both IL-4 and IL-13 is highly effective in AD patients. Dupilumab is expected to be the first biologic being launched for AD treatment after having shown efficacy in several clinical trials (57). However, these analyses clearly highlight an intrinsic failure to terminate IL-4- and TLR2-driven chronic self-perpetuating AD as prominent feature of AD pathogenesis.

**Figure 3:**
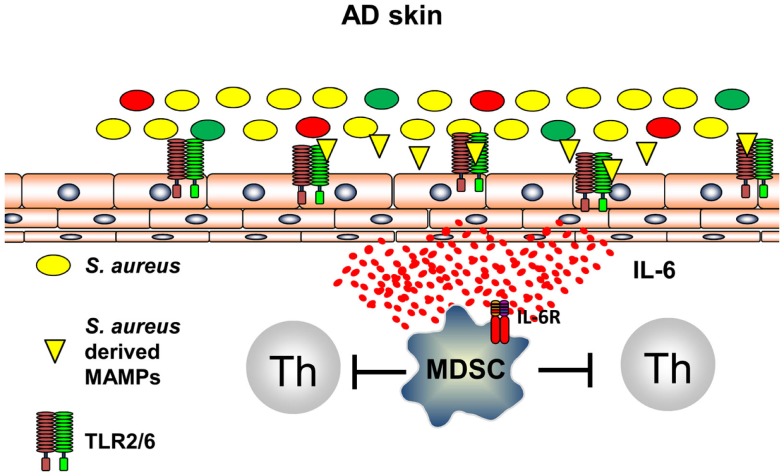
***S. aureus*-derived lipoproteins induce MDSC**. TLR2/6 heterodimers are expressed on skin-resident cells. Binding of diacylated bacterial lipoproteins induces IL-6 secretion leading to accumulation and activation of MDSC in the skin. T cell activation is vigorously suppressed by a NO-dependent mechanism.

It can be anticipated that all types of inflammation, including those of anti-infectious immune responses, induce immune pathways that are capable to terminate inflammation and to prevent exaggerated and self-perpetuating potentially harmful courses of inflammation. Recently, it could be shown that innate immune recognition of *S. aureus* induces, following TLR2-induced inflammation, immune suppression by induction of myeloid-derived suppressor cells (MDSC) in a mouse model of AD (58). *S. aureus-*derived lipoproteins binding exclusively to the TLR2/6 heterodimer on skin-resident cells trigger IL-6 secretion that leads to activation and recruitment of CD11b^+^ Gr1^+^ MDSC into the skin (Figure 3). These CD11b^+^ Gr1^+^ MDSC efficiently suppressed T cell recall responses in the skin by an iNOS-dependent pathway. These MDSC, however, fail to suppress AD inflammation, but rather inhibit anti-infectious immune responses allowing *S. aureus* to further spread and drive AD and possibly also herpes viruses to mount a dangerous AD complication, eczema herpeticum (58). These investigations show that innate immune recognition of pathogenic bacteria on the skin induces an anti-inflammatory pathway presumably to limit damage induced by inflammatory responses. Distinct temporal and spatial distributions may coordinate these different and counter regulatory immune responses and understanding underlying mechanisms of attempts to prevent or resolve inflammation are important areas of research to identify new targets of treatments. Some of these mechanisms may be identified by the analyses that focus on the skin microbiome and immune consequences derived of the communication between the microbiome, the skin, and the cutaneous immune system.

## Skin Microbiome

The skin is constantly colonized by myriads of bacteria with approximately 10^6^ bacteria homing per square centimeter resulting in a total of 10^10^ bacteria covering the whole skin (59). The composition and distribution of the cutaneous microbiota have been deciphered in yet unknown details using deep sequencing techniques (60, 61). These techniques revealed a much more diverse microbiota as previously anticipated and detected using culture-based methods (61). The majority of bacteria identified by 16S ribosomal RNA sequencing can be assigned to four major phyla: Actinobacteria, Firmicutes, Bacteroidetes, and Proteobacteria (62). Importantly, 16S rRNA sequencing also detected Gram-negative species at dry skin areas, which were previously seen as contaminants but not residents using culture-based techniques (63).

Examining different skin sites, it was found that the skin microbiota differs between topographical locations (60). The colonization of bacteria on the different areas of human skin is largely dependent on the physiology of the respective skin site. Whether the area is predominantly “moist,” “dry” or “sebaceous” impacts the respective microbiota to a large extent. At moist areas, *Staphylococcus spp*. and *Corynebacterium spp*. are the most abundant bacterial communities detected (59, 60). The highest diversity of phylotypes can be observed at dry skin areas like forearms, buttocks, and part of the hands with multiple species from the phyla Actinobacteria, Firmicutes, Bacteroidetes, and Proteobacteria being detected.

In contrast, sebaceous sites have the lowest diversity of phylotypes with a predominance of *Propionibacterium spp*. confirming culture-based approaches showing that *Propionibacterium spp*. are commensals in areas rich in the “pilosebaceous units” consisting of hair, hair follicle, and sebaceous gland (60).

Comparing interpersonal and intrapersonal variations of the skin microbiota at distinct topographical sites, it was shown that interpersonal variations are less than anticipated indicating that the colonized niche and its physiological features are a much stronger determinant for composition of the bacterial colonization than genetic variations of the individuals investigated (59, 62). In addition, the microbiota of one individual of, e.g., some sebaceous sites that share the same ecological features display a large similarity, further supporting the concept of cutaneous habitats determining the compositions of microbes by the cutaneous milieu.

Microbial colonization of the skin is established early in life. With birth, transition of the newborn’s surfaces derived from a sterile uterine milieu then exposed to a microbial-rich environment occurs. Thus, immediately after birth the skin of the newborn is being colonized (64). The mode of delivery has been shown to play a major impact on the composition of the skin microbiota of the newborn. Following vaginal delivery, the skin microbiota resembles the mother’s vaginal microbiota. In contrast, following cesarean delivery, the skin microbiota of the mother and the skin microbiota of the newborn show striking differences (64). The impact of these observations on the composition of the individual microbiota and the infant’s health is largely unknown, but consequences for immune responses have been postulated. Interestingly, the composition of the skin microbiota of 1 to 3-month-old children did not differ in regard to mode of delivery (65), whereas other studies demonstrated a striking stability of individual microbiota over time. Indeed, the microbiome of adults is maintained over time whereas the microbiome of children shows less stability and even an increase in microbial diversity over time (65). The consequences of individual microbiota developing at different time points for immune responses, immune tolerance development, and the “readiness” for defense are still a matter of debate. Whether disturbances and dysbiosis during the process of microbiota development influence susceptibility to skin diseases associated with dyscolonization of the skin remains to be established.

## Functional Consequences of the Skin Microbiome

The studies of the intestinal and the cutaneous microbiome are for the most part descriptive in nature and associations of certain microbiota or of compositions within detected microbiota with diseases are increasingly being detected. However, functional consequences still need to be proven, underlying mechanisms of diseases need firm linkage to microbial exposure, and pathways of translating surface exposure to microbes into immune responses and memory still need to be established. Only few mechanisms of how the skin microbiome or specific microorganisms contribute to cutaneous immune homeostasis have been identified so far. Importantly, functional studies need to also involve pre-clinical analyses in disease models. However, animals with fur may not follow the same rules and algorithms as human skin in the absence of fur.

Much attention has been given to consequences of cutaneous exposure to s*taphylococci* with the assumption of *Staphylococcus epidermidis* being the “good” part of the microbiome and *S. aureus* being the “bad” causing infections and disease. Indeed, cutaneous colonization of severe barrier-disrupted murine skin with *S. aureus* leads to expression of IL-1β, IL-6, and TNFα lasting for up to 6 days demonstrating a pivotal role for *S. aureus* in promoting skin inflammation in susceptible hosts (66). After skin wounding and subsequently initiated inflammation due to TLR3-mediated detection of damaged cells, the skin commensal *Staphylococcus epidermidis* was identified to mediate resolution of cutaneous inflammation. Binding of *Staphylococcus epidermidis* derived lipoteichoic acid to TLR2 on keratinocytes was crucial in mediating this anti-inflammatory function via a TRAF1 dependent pathway (67). Recently, it was shown that recognition of *S. epidermidis* by the innate immune system profoundly shapes adaptive immune responses (68). Colonization of the skin with *S. epidermidis* activates antigen-specific T cells secreting IL-17 and IFN-γ in an IL-1R- and MyD88-dependent manner at steady-state conditions (68). Lack of commensals lead to a failure of induction of dermal IL-17- and IFN-γ-producing αβ T cells but raised the numbers of FoxP3^+^ regulatory T cells in the skin. To identify functional consequences of cutaneous *S. epidermidis* exposure, a model of Leishmania infection was applied showing that in the absence of sufficient immune sensing of *S. epidermidis*, Leishmania infection could not be controlled sufficiently (68). Further analysis of the cellular pathways involved in this process showed that IL-17-producing T cells induced by *S. epidermidis* belong to the CD8+ subset displaying a unique and previously unknown role for these T cells in providing immune defense mechanisms in the skin (69). Thus, colonization of the skin with the commensal *S. epidermidis* provides innate immune signals to set up a functional threshold for adaptive immunity to establish pathogen control. Adaptive immune responses induced by the microbiota are required not only to fight pathogens but also to control colonization of the skin with commensals as in the absence of adaptive immunity commensal bacteria were detected in the local lymph nodes indicating microbial invasion (70). Skin microbiota therefore seems to induce a feedback loop to control microbial colonization with commensals to maintain epithelial integrity and immune homeostasis.

Changes of the skin microbiota composition might therefore contribute to cutaneous inflammation seen in various skin diseases (71). Detailed analysis of the cutaneous microbiota of AD patients demonstrated dramatically reduced diversity of the microbiome analyzed from acute flares presenting at the antecubital and popliteal crease (1). Instead of microbiota diversity overabundance of *S. aureus* and *S. epidermidis* was detected and correlated with disease severity confirming previous observations demonstrating *S. aureus* colonization and worsening of AD (1, 72). Of note, the “hen and egg problem” is not solved. Loss of microbiome diversity leading to skin flares or skin flares orchestrating dramatically reduced diversity of the microbiome are both possible scenarios. While for humans, the latter was thought to be the more likely cascade of events, new insights were brought forward by a mouse model.

A new model to study these changes from humans in detail including possibly causal relations was established most recently: mice lacking epidermal ADAM17 (*Adam17*^ΔSox9^, *A17*^ΔKC^) exhibit skin barrier disruption with enhanced transepidermal water loss (TEWL) and subsequently develop eczematous lesions with a lymphocytic infiltrate resembling human AD (73, 74). The skin microbiome of mice with disrupted barrier function due to epidermal deficiency of ADAM17 (*Adam17*^ΔSox9^) was indistinguishable from wildtype littermates in the first 2 weeks after birth but showed decreased bacterial diversity and an abundance of *Staphylococcus spp*. and *Corynebacterium spp*. compared to wildtype controls starting 4 weeks after birth (74). Administration of antibiotics in *Adam17*^ΔSox9^ mice targeting *S. aureus* prevented the development of eczematous lesions and TEWL as well as cytokine production by CD4+ T cells and an increased number of skin-infiltrating T cells. Moreover, the microbiome showed a higher diversity – despite the use of antibiotics – and was comparable to the one of wildtype controls. In crossover experiments, antibiotic depletion of *S. aureus* in *Adam17*^ΔSox9^ mice resulted in reduction of eczematous lesions, reduced TEWL, attenuation of skin-infiltrating T cells, and reversal of dysbiosis. By contrast, withdrawal of antibiotics in previously administered *Adam17*^ΔSox9^ mice resulted in development of eczema, enhanced TEWL, increased skin-infiltrating T cells, and severe dysbiosis with excessive *S. aureus* colonization (74). These data clearly demonstrate that a shift of the microbiome resulting in dysbiosis with reduced microbial diversity and overrepresentation of *S. aureus* strikingly contributes to development of cutaneous inflammation and acute atopic flares in both mice with disrupted barrier and susceptible humans (1, 74). Thus enhancing the diversity of the microbiome to support colonization with putative non-pathogenic beneficial bacteria and/or targeting *S. aureus* colonization may be a therapeutic strategy in treatment of AD.

## Resolution and Prevention of Atopic Skin Inflammation

While functional analysis of the skin microbiome is currently in its beginnings, much more details on the functional properties of the gut microbiota were gathered (75, 76). At steady-state conditions, IL-17A- and IFN-γ-producing T cells can be found in the gut-associated lymphoid tissue (GALT) and are required for prevention of inflammation by intestinal pathogens (77). Induction of these T cell populations has been shown to be dependent on the presence of the gut microbiota because in germ-free mice their numbers are significantly reduced (77, 78). This situation closely resembles what was found in the skin, namely turning off the local immune response by commensals to avoid inflammation by pathogens (71, 79). In contrast to the skin, FoxP3^+^ regulatory T cells (Tregs) are abundantly present in the GALT especially lining the lamina propria and Treg induction is dependent on the intestinal microbiota (79, 80). Several distinct mechanisms for induction of these regulatory T cells by the microbiota have been elucidated. Polysaccharide A (PsA) from Gram-negative *Bacteroides fragilis* has been shown to bind to TLR2 and to exert immune modulatory capacities in murine models of intestinal inflammation (81). The regulatory effects of PsA are due to induction of FoxP3^+^ iTregs and IL-10 secretion. Furthermore PsA is critical for maintaining immune homeostasis in the gut epithelium (81). A mixture of non-pathogenic *Clostridium spp* lacking toxins and virulence factors was identified to induce FoxP3^+^ Tregs in a TGF-β dependent manner preventing experimentally induced intestinal inflammation (82, 83). Whether analogous approaches performed in animal models for the gut are also feasible to attenuate skin inflammation was not yet investigated.

**Figure 4:**
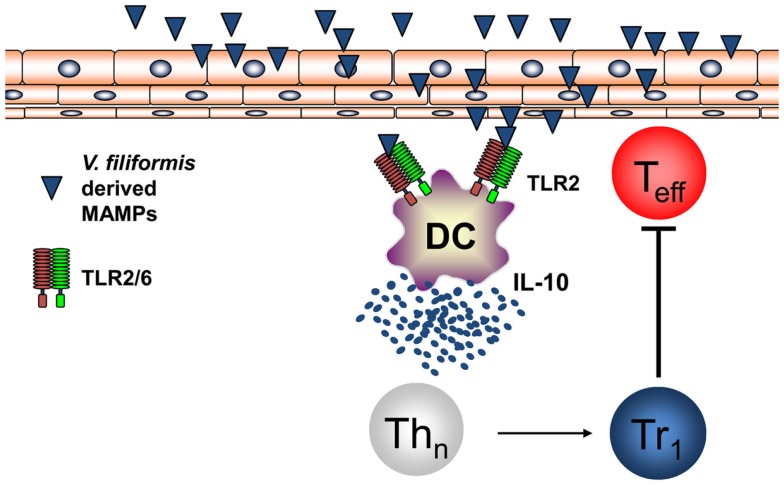
**Non-pathogenic bacterium *V. filiformis-*derived MAMPs induce tolerogenic DC and Tr1 cells**. *V. filiformis-*derived MAMPs activate DC to produce IL-10 via a TLR2-dependent mechanism. DC-derived IL-10 is required to subsequently polarize naïve T helper cells into a Tr1 phenotype characterized by low IFN-γ and high IL-10 secretion. Vf-induced Tr1 cells efficiently block effector T cells (T_eff_) demonstrating regulatory function for attenuating skin inflammation.

Recently, it was shown by investigating humans that the diversity of the environmental microbiota and the prevalence of atopic diseases are interrelated and in atopic individuals a significant reduced diversity of Gammaproteobacteria at their surroundings and on their skin could be found (84). Relative abundance of Gammaproteobacteria was correlated with IL-10 secretion by PBMCs of human healthy individuals while this IL-10 secretion was lacking in atopic patients. Investigations on the level of genus revealed that the reduced frequency of Gram-negative *Acinetobacter* best correlated with diminished IL-10 production in atopic individuals (84). Moreover, it was shown that heat-inactivated *Acinetobacter Iwoffi* induces IL-10 production in DC and primary human keratinocytes *in vitro* and when applied intradermally (85). This work extends investigations that showed that *Acinetobacter Iwoffi* exposure of pregnant mice avoids allergic asthma development in their off springs (86). These findings highlighting a possibly beneficial role for Gram-negative bacteria both for prevention of atopic diseases and for maintaining skin microbiota propose to investigate new therapeutic strategies extending the regulation of the skin microbiome beyond the focus on Gram-positive bacteria such as *S. epidermidis*. We performed the first proof-of-concept RCT in humans to investigate whether signals derived from non-pathogenic Gram-negative bacteria may mediate beneficial effects for the skin with mild AD. Therefore, we initiated a randomized double-blind placebo-controlled clinical trial on AD patients using a lysate from Gram-negative *Vitreoscilla filiformis*. Groups of patients with AD received either vehicle cream or this vehicle cream supplemented with a lysate of *V. filiformis* for 29 days. A first analysis following 15 days of treatment already detected a significant change in the group treated with the *V. filiformis* supplemented cream. Following 29 days of treatment with *V. filiformis*-derived substances, a significant reduction of disease activity in patients receiving topical therapy with a cream containing *V. filiformis* was observed, moreover, intergroup comparison revealed a significant difference between groups demonstrating clinical efficacy of immune signals derived from Gram-negative *V. filiformis* (87). Consecutive work focused on underlying mechanisms mediating immune modulation induced by immune signals derived from Gram-negative *V. filiformis*. To be able to analyze this mechanism of action possibly inducing tolerogenic immune responses effective on the skin, a mouse model for AD was utilized. Topical application of a lysate of *V. filiformis*, the Gram-negative, non-pathogenic bacterium, was shown to suppress skin inflammation in Th2-dominated hypersensitivity in the AD-prone NC/Nga mice (88). To unravel the underlying mechanisms, a series of *in vitro* and *in vivo* experiments was performed. It could be shown that *V. filiformis* lysate predominantly induces IL-10-producing DC in a TLR2-and MyD88-dependent manner. Priming of naïve T cells with DC activated by *V. filiformis* lead to the induction of Tr1 cells, which are characterized by their cytokine profile with high IL-10 and low IFN-γ levels (88, 89). The induction of IL-10^high^ IFN-γ^low^ Tr1 cells by innate immune signals from *V. filiformis* was dependent on DC-derived IL-10 and TLR2-signaling in DC. *V. filiformis* induced Tr1 cells had the capacity to suppress effector T cell proliferation and function demonstrating their regulatory function (Figure 4). Enhanced IL-10 production, reduced IFN-γ levels and diminished T cell proliferation in skin draining lymph nodes of mice topically treated with *V. filiformis* lysate compared to controls was observed demonstrating *in vivo* functionality of this approach (88). These data demonstrate that cutaneous exposure to innate immune signals derived of non-pathogenic Gram-negative bacteria is sufficient to induce long-lasting immune tolerance by induction of IL-10, that limited cutaneous exposure is capable to induce also systemic immune modulation, and that indirect supplementation of IL-10 lacking in atopic individuals by innate immune signals from non-pathogenic Gram-negative bacteria may be a feasible therapeutic approach to stabilize both the cutaneous barrier and immune homeostasis.

## Summary

T helper cells play a key role in eliciting and maintaining AD inflammation. Therefore, modulating T cell-elicited immune responses is a promising therapeutic approach. Th1- and Th1/Th17-mediated skin inflammation leading to psoriasis or cutaneous delayed-type hypersensitivity reactions could be treated successfully by immune deviation approaches targeting T helper cell polarization and inducing immune deviation (25, 26, 28). The contribution of various T helper cell subsets (Th2, Th1 Th22, maybe Th17) to AD pathophysiology precludes application of this successful principle as it could be detrimental and result in worsening of disease severity. Thus, induction of tolerogenic immune responses promises to be a feasible strategy. Innate immune recognition of specific components or bacteria derived from the gut microbiota has been shown to induce tolerance based on induction of tolerogenic DC, regulatory T cells, and anti-inflammatory cytokines. This approach could by successfully applied to dampen AD inflammation by activating DC with innate immune signals of non-pathogenic bacteria resulting in induction of tolerogenic DC, priming of regulatory Tr1 cells, and attenuation of cutaneous inflammation. After identification of diminished bacterial diversity in the skin microbiome of atopic individuals with consecutive loss of anti-inflammatory and tolerogenic IL-10, substitution of these tolerance promoting innate immune signals using microbes or microbial components is a new and promising therapeutic strategy.

## Conflict of Interest Statement

The authors declare that the research was conducted in the absence of any commercial or financial relationships that could be construed as a potential conflict of interest.
